# Vitamin D_3_ Supplementation Increases Long-Chain Ceramide Levels in Overweight/Obese African Americans: A Post-Hoc Analysis of a Randomized Controlled Trial

**DOI:** 10.3390/nu12040981

**Published:** 2020-04-02

**Authors:** Li Chen, Yanbin Dong, Jigar Bhagatwala, Anas Raed, Ying Huang, Haidong Zhu

**Affiliations:** 1 Georgia Prevention Institute, Department of Medicine, Medical College of Georgia, Augusta University, Augusta, GA 30912, USA; lichen1@augusta.edu (L.C.); ydong@augusta.edu (Y.D.); yihuang@augusta.edu (Y.H.); 2 Department of Medicine, Medical College of Georgia, Augusta University, Augusta, GA 30912, USA; JBHAGATWALA@augusta.edu (J.B.); ARAED@augusta.edu (A.R.)

**Keywords:** vitamin D, ceramide, sphingolipids, randomized clinical trial

## Abstract

Sphingolipid metabolism plays a critical role in cell growth regulation, lipid regulation, neurodevelopment, type 2 diabetes, and cancer. Animal experiments suggest that vitamin D may be involved in sphingolipid metabolism regulation. In this study, we tested the hypothesis that vitamin D supplementation would alter circulating long-chain ceramides and related metabolites involved in sphingolipid metabolism in humans. We carried out a post-hoc analysis of a previously conducted randomized, placebo-controlled clinical trial in 70 overweight/obese African-Americans, who were randomly assigned into four groups of 600, 2000, 4000 IU/day of vitamin D_3_ supplements or placebo for 16 weeks. The metabolites were measured in 64 subjects (aged 26.0 ± 9.4 years, 17% male). Serum levels of *N*-stearoyl-sphingosine (d18:1/18:0) (C18Cer) and stearoyl sphingomyelin (d18:1/18:0) (C18SM) were significantly increased after vitamin D_3_ supplementation (*p*s < 0.05) in a dose–response fashion. The effects of 600, 2000, and 4000 IU/day vitamin D_3_ supplementation on C18Cer were 0.44 (*p* = 0.049), 0.52 (*p* = 0.016), and 0.58 (*p* = 0.008), respectively. The effects of three dosages on C18SM were 0.30 (*p* = 0.222), 0.61 (*p* = 0.009), and 0.68 (*p* = 0.004), respectively. This was accompanied by the significant correlations between serum 25-hydroxyvitamin D_3_ [25(OH)D] concentration and those two metabolites (*p*s < 0.05). Vitamin D_3_ supplementations increase serum levels of C18Cer and C18SM in a dose–response fashion among overweight/obese African Americans.

## 1. Introduction

Sphingolipids are bioactive lipids and key components of cell membranes, which exert critical roles in signal transduction [1]. Sphingolipids were first discovered in the 1880s in the brain [2], the metabolites of which have been found as important molecules that regulate cell growth, apoptosis, proliferation, and migration, and are involved in the pathologies of a variety of diseases, especially inflammation, insulin sensitivity [3,4], and cancer [5,6].

Ceramide (Cer), composed of a sphingosine backbone *N*-acylated with different fatty acyl-CoA [7], is a multifunctional central molecule in sphingolipid metabolism. The biological properties and functions of Cers have been studied extensively in recent years. Cer is a powerful tumor suppressor [8], which triggers a variety of tumor-suppressive and anti-proliferative cellular programs such as apoptosis, autophagy, senescence, and necroptosis [9]. Cer is also beneficial for the early growth and development of neuronal cells [10]. 

Vitamin D deficiency is associated with a higher risk of developing type 2 diabetes (T2D). Studies suggest that vitamin D supplementation may improve glycemic controls and insulin sensitivity [11,12]. Sphingolipid signaling is also involved in the development of T2D [3,4]. Ceramides are suggested to be independent antagonists to insulin signaling [13] and induce insulin resistance [14]. But ceramide species exert different effects depending on the chain-lengths of the fatty acid bound to the sphingosine backbone [15]. *N*-palmitoyl-sphingosine (d18:1/16:0) (C16Cer) is the principal mediator of obesity-related insulin resistance [16,17]. Other studies also found *N*-stearoyl-sphingosine (d18:1/18:0) (C18Cer) to be positively related to insulin resistance [18], and treatment to improve insulin sensitivity would reduce plasma level of C18Cer as well [19]. Animal experiments suggest that vitamin D may be involved in sphingolipid metabolism regulation [20]. However, evidence in humans is scarce. To the best of our knowledge, only one randomized controlled trial (RCT) has been conducted, which reports that vitamin D supplementation regulates sphingolipid metabolism in a European population with type 2 diabetes [15]. Therefore, in this study, for the first time, we tested the hypothesis that vitamin D supplementation would alter the sphingolipid metabolism in a dose–response fashion among overweight/obese African Americans by a post-hoc analysis of a previously conducted RCT.

## 2. Materials and Methods

### 2.1. Participants

As previously described [21], 70 overweight/obese apparently healthy African-Americans residing in Augusta, Georgia, and surrounding areas, who were vitamin D insufficient, were randomized into a double-blinded and placebo-controlled trial (NCT01583621) during December 2011 and November 2012 (Supplementary Figure S1). Inclusion criteria were self-reported African-American race, aged between 13–45 years, overweight or obese (body mass index [BMI] ≥ 25 kg/m^2^ for adults and ≥85th percentile for age and sex otherwise), no pregnancy, no known acute or chronic illnesses (e.g., diabetes or cancer), no use of any prescription medications, birth control pills, herbal, multi-vitamin or mineral supplementations, and suboptimal vitamin D status (serum 25-hydroxyvitamin D_3_ (25(OH)D) concentrations of ≤20 ng/mL (50 nmol/L)). Six participants were lost during the follow-up. Informed consent was obtained from the adults and the guardians of adolescents. The study was approved by the institutional review board (IRB) at Augusta University.

### 2.2. Randomization and Treatments

The participants were randomly assigned to any one of the four groups 18,000 IU/month (~600 IU/day), 60,000 IU/month (~2000 IU/day), 120,000 IU/month (~4000 IU/day) of vitamin D_3_ or placebo, and the interventional capsules were provided to the participants by supervised dosing for 16 weeks to maximize compliance. The vitamin D_3_ and placebo capsules were provided by the Bio-Tech Pharmacal, Fayetteville, AR, and the AU clinical research pharmacy generated the randomization codes and dispensed the study capsules. The AU clinical pharmacy maintained the randomization codes until the end of the study and did not have any direct role in the data collection [21].

### 2.3. Measurements and Laboratory Assessments

Height and weight were obtained according to standard procedures and BMI was calculated as weight (kg) divided by height (m^2^). Fasting blood samples were collected at baseline and 16 weeks, which were frozen and stored at −80 °C until assayed. Serum 25(OH)D concentrations were measured using enzyme immunoassay (Immunodiagnostic Systems, Fountain Hills, AZ, USA). The intra- and inter-assay coefficients of variation (CV) were 5.6% and 6.6%, respectively. Our laboratory is certified by the vitamin D external quality assessment scheme (DEQAS), an international program monitoring accuracy of 25(OH)D measurements. Hemoglobin A1c was measured by Clinical Pathology Labs (MCG Health, Inc., Augusta, GA, USA). 

### 2.4. LC-MS/MS Analysis

Sphingolipid metabolites were analyzed using LC-MS/MS methods in a global fashion. A total of 128 fasting serum samples at baseline and post 16-week intervention from 64 participants who completed the study were processed by Metabolon platform (METABOLON, INC. NC, USA). The sample preparation process was carried out using the automated MicroLab STAR^®^ system (Hamilton Company, Salt Lake City, UT, USA). The extract was divided into five fractions: two for analysis by two separate reverse phase (RP)/UPLC-MS/MS methods with positive ion mode electrospray ionization (ESI), one for analysis by RP/UPLC-MS/MS with negative ion mode ESI, one for analysis by HILIC/UPLC-MS/MS with negative ion mode ESI, and one sample was reserved for backup. All methods utilized a Waters ACQUITY ultra-performance liquid chromatography (UPLC) and a Thermo Scientific Q-Exactive high resolution/accurate mass spectrometer interfaced with a heated electrospray ionization (HESI-II) source and Orbitrap mass analyzer operated at 35,000 mass resolution. Raw data were extracted, peak-identified, and quality control (QC) processed using Metabolon’s hardware and software. Compounds were identified by comparison to library entries of purified standards or recurrent unknown entities. Peaks were quantified using area-under-the-curve.

### 2.5. Statistical Analysis

The general characteristics of the subjects are presented as mean ± standard deviation (SD) for continuous variables and N (%) for categorical variables. The normality of each continuous variable was tested based on a combination of test statistics of skewness and kurtosis. Baseline group differences were determined by analysis-of-variance (ANOVA) for normally distributed variables or by the Kruskal–Wallis test, otherwise. Group differences in proportions at baseline were tested by Fisher’s exact test. 

Before statistical analysis, sphingolipid metabolites data were log-transformed and standardized to unit variance and zero mean. Mixed-effects models for repeated measures were used in an intention-to-treat analysis using all available data. Models for levels of metabolites included the fixed effects of intervention groups (placebo, 600, 2000, or 4000 IU/day), measurement time (baseline or posttest), and their interaction. The models were also adjusted for age, gender, and BMI. Standardized β coefficients were presented. We also tested the associations between serum 25(OH)D concentrations and the sphingolipids, and the associations of sphingolipids with BMI and A1c. A *p*-value < 0.05 was considered statistically significant. All statistical analysis was performed using Stata version 12.0 (College Station, TX 77845, USA).

## 3. Results

### 3.1. General Characteristics

The demographics of the participants are shown in Table 1. Sixty-four participants (aged 26.0 ± 9.4 years, 17% are male) were included in the analysis. There was no significant difference among the four groups regarding age, sex, BMI, and 25(OH)D at baseline (*p*s > 0.05). 

### 3.2. Effects of Vitamin D_3_ Supplementation on Sphingolipid Metabolites

**Figure 1:**
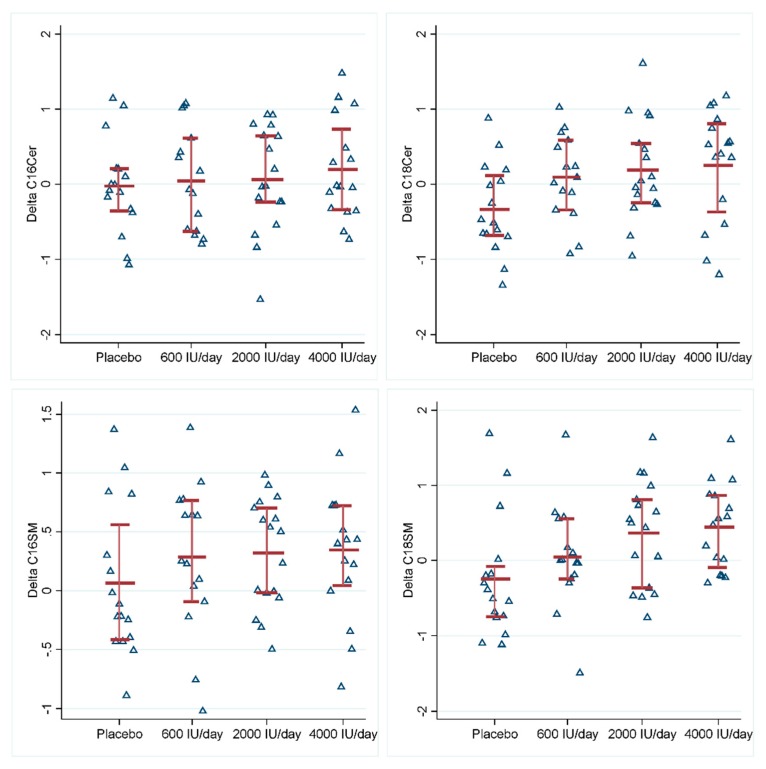
Effects of vitamin D_3_ supplementation on serum ceramide levels and sphingomyelin levels. The upper left is C16Cer, upper right is C18Cer, lower left is C16SM, and lower right is C18SM. Y-axis is the change in standardized levels of ceramide or sphingomyelin. Red lines indicate 25 percentile, mean and 75 percentile of standardized levels of ceramide or sphingomyelin in each group. Abbreviations: C16Cer, *N*-palmitoyl-sphingosine (d18:1/16:0); C18Cer, *N*-stearoyl-sphingosine (d18:1/18:0); C16SM, palmitoyl sphingomyelin (d18:1/16:0); C18SM, stearoyl sphingomyelin (d18:1/18:0).

Serum levels of long-chain ceramides C18Cer and C18SM significantly increased after vitamin D_3_ supplementation in a dose–response fashion (*p*s < 0.05) (Table 2, Figure 1, Supplementary Table S1). The effects of 600, 2000, and 4000 IU/day vitamin D_3_ supplementation on C18Cer were 0.44 (*p* = 0.049), 0.52 (*p* = 0.016), and 0.58 (*p* = 0.008), respectively. Similarly, the effects of three dosages of vitamin D_3_ supplementation on C18SM were 0.30 (*p* = 0.222), 0.61 (*p* = 0.009), and 0.68 (*p* = 0.004), respectively. Their degradation product sphingosine decreased after vitamin D_3_ supplementation, while *N*-stearoyl-sphinganine (d18:0/18:0) (C18dhCer) as a precursor of C18Cer increased after the supplementation, but the changes were not statistically significant (*p*s > 0.05) (Supplementary Figures S2 and S3). All the other metabolites remained unchanged (Table 2). Changes in C18Cer and C18SM levels were also significantly associated with changes in serum 25(OH)D concentration, regardless of treatments (Table 3, Supplementary Figures S4–S7). At baseline, serum 25(OH)D concentration was inversely associated with C16SM (*p* = 0.004) (Supplementary Table S2).

### 3.3. Associations of the Changes in Sphingolipids with the Changes in BMI and A1c

The changes in sphingolipids were associated with the changes in BMI and A1c using mixed-effects linear regressions. The changes in C18dhCer, sphingosine, and S1P were positively associated with the changes in A1c (*p*s < 0.05), the change in C18Cer and C18dhCer were associated with the changes in BMI (*p*s < 0.005) (Table 4).

## 4. Discussion

The present study shows that vitamin D_3_ supplementations increase serum levels of long-chain *N*-stearoyl-sphingosine (d18:1/18:0) (C18Cer) and stearoyl sphingomyelin (d18:1/18:0) (C18SM) in a dose–response fashion among overweight/obese African Americans. 

Cer is the central hub of sphingolipid metabolism (Supplementary Figure S8). The increase of sphingolipid metabolite C18Cer by vitamin D_3_ supplementation observed in our RCT is consistent with the previous report [15]. Several studies suggest that sphingolipids such as ceramides influence metabolic pathways and are involved in the development of T2D [3,4]. 

C18Cer is also associated with neurodegeneration. In the central nervous system (CNS), the most highly expressed ceramide synthase is CerS1, which synthesizes C18Cer. CerS1 is particularly present in neurons of neocortex, hippocampus, and cerebellum [22]. Molecular scanning of individual ganglioside molecular species showed a significant reduction of C18Cer-containing gangliosides in patients with Alzheimer’s disease [23]. Impairment of C18Cer biosynthesis underlies neurodegeneration and causes novel progressive myoclonus epilepsy in humans [22]. Vitamin D deficiency is associated with neurodegenerative diseases, such as Alzheimer’s disease [24,25], dementia [26], and cognitive disturbances [27]. Therefore, it is plausible that C18Cer may link vitamin D deficiency to neurodegeneration disease [25]. Vitamin D_3_ supplementation increased C18Cer in the present study, which may help to slow down the progression of the neurodegeneration process. 

C18SM, another vitamin D associated metabolite, is a reservoir of other sphingolipids [28], and itself also plays an important role in cell membrane formation and plasma lipoprotein metabolism. Studies show that SM consumption may reduce cholesterol absorption and improve lipid profiles [29,30]. Altered SM is also involved in neurodevelopment [31]. An RCT found that SM-fortified milk had a positive association with the neurobehavioral development of very low birth weight infants during infancy [31]. We showed that vitamin D_3_ supplementation increased C18SM, which may be beneficial to neurodevelopment.

Evidence relating vitamin D to sphingolipids is limited, while hypotheses can be speculated. A study found that silencing vitamin D receptor (VDR) caused decreased transcription of ceramide glucosyltransferase (GlcT-1), which demonstrated that VDR is critical for sphingolipid production and barrier formation [20]. Vitamin D metabolites were also shown to be able to activate the SM pathway and induce an increase in cellular Cer concentration [32]. Vitamin D induces SM hydrolysis, which seems to be a ubiquitous pathway to generate Cer [33,34].

Sphingolipids metabolism plays a critical role in obesity. To the best of our knowledge, this is the first study examining the effects of vitamin D_3_ supplementation on sphingolipid metabolism in overweight/obese populations. We also target overweight/obese African Americans that this ethnic group is prone to suboptimal vitamin D status and higher cardiovascular risk [35], and adiposity is considered to sequester vitamin D [36]. Moreover, we are the first to investigate the effects of vitamin D supplementation on sphingolipids in a dose–response fashion by providing three different doses. However, the sample size of our study is modest. Further large-scale studies are needed to validate our results. In addition, our findings were based on overweight/obese African Americans and may not be generalizable to other populations. At last, this is a post-hoc analysis of a randomized controlled trial, such that there is a likelihood of either false-positive or false-negative results.

## 5. Conclusions

Vitamin D supplementations may regulate sphingolipid metabolism, and increase serum levels of C18Cer and C18SM in a dose–response fashion, which are found to be critical in cell growth, lipid metabolism, neurodevelopment, and cancer. 

## Figures and Tables

**Table 1:** Baseline characteristics among different groups *.

Characteristics	Total	Groups				
		Placebo (N = 16)	600 IU/day (N = 15)	2000 IU/day (N = 17)	4000 IU/day (N = 16)	*p*-Values
Age (year)	26.0 ± 9.4	27.9 ± 10.4	26.3 ± 9.8	24.5 ± 8.5	25.5 ± 9.6	0.833
Male (N)	11 (17)	4 (25)	2 (13)	3 (18)	2 (13)	0.843
BMI (kg/m^2^)	35.7 ± 7.2	36.2 ± 8.1	34.6 ± 5.6	37.1 ± 7.7	34.8 ± 7.3	0.636
Obese (N)	51 (80)	12 (75)	12 (80)	14 (82)	13 (81)	0.974
A1c (%)	5.4 ± 0.4	5.4 ± 0.5	5.4 ± 0.5	5.3 ± 0.5	5.5 ± 0.3	0.422
25(OH)D (nmol/L)	36.9 ± 11.3	39.7 ± 14.7	35.0 ± 7.7	39.8 ± 10.7	32.9 ± 10.3	0.276

* Statistics display as mean ± SD for continuous variables, and N (%) for categorical variables. Baseline group differences of continuous variables were determined by ANOVA for normally distributed variables or by the Kruskal–Wallis test, otherwise. Fisher’s exact test was carried out on categorical variables. BMI, body mass index; 25(OH)D, 25-hydroxyvitamin D_3_.

**Table 2:** Adjusted associations between sphingolipids and vitamin D_3_ supplementation *.

Metabolites.	600 IU/day		2000 IU/day		4000 IU/day	
	β (95% CI)	*p*	β (95% CI)	*p*	β (95% CI)	*p*
C16Cer	0.06 (−0.40, 0.53)	0.791	0.09 (−0.36, 0.54)	0.702	0.22 (−0.23, 0.54)	0.336
C18Cer	0.44 (0.00, 0.87)	0.049	0.52 (0.10, 0.94)	0.016	0.58 (0.15, 1.00)	0.008
C16dhCer	−0.06 (−0.57, 0.46)	0.830	−0.22 (−0.71, 0.28)	0.369	−0.00 (−0.51, 0.50)	0.988
C18dhCer	0.28 (−0.33, 0.90)	0.363	0.54 (−0.05, 1.15)	0.071	0.39 (−0.21, 1.00)	0.200
Sphingosine	−0.09 (−0.88, 0.71)	0.833	−0.27 (−1.03, 0.50)	0.499	−0.35 (−1.13, 0.43)	0.382
S1P	−0.17 (−0.86, 0.53)	0.638	0.26 (−0.42, 0.93)	0.460	−0.42 (−1.10, 0.27)	0.232
C16SM	0.21 (−0.19, 0.62)	0.299	0.26 (−0.13, 0.65)	0.198	0.28 (−0.11, 0.68)	0.160
C18SM	0.30 (−0.18, 0.76)	0.222	0.61 (0.15, 1.06)	0.009	0.68 (0.22, 1.15)	0.004

* Mixed-effects models were adjusted for age, sex, and BMI. Levels of metabolites were standardized. Abbreviations: C16Cer, *N*-palmitoyl-sphingosine (d18:1/16:0); C18Cer, *N*-stearoyl-sphingosine (d18:1/18:0); C16dhCer, *N*-palmitoyl-sphinganine (d18:0/16:0); C18dhCer, *N*-stearoyl-sphinganine (d18:0/18:0); S1P, sphingosine 1-phosphate; C16SM, palmitoyl sphingomyelin (d18:1/16:0); C18SM, stearoyl sphingomyelin (d18:1/18:0).

**Table 3:** Adjusted associations between the changes in sphingolipids and the changes in 25(OH)D concentrations *.

Metabolites	25(OH)D	
	β (95% CI)	*p*
C16Cer	0.13 (−0.31, 0.57)	0.570
C18Cer	0.44 (0.02, 0.86)	0.041
C16dhCer	−0.01 (−0.51, 0.50)	0.973
C18dhCer	0.39 (−0.16, 0.93)	0.161
Sphingosine	0.08 (−0.68, 0.83)	0.841
S1P	−0.10 (−0.77, 0.57)	0.758
C16SM	0.25 (−0.12, 0.61)	0.179
C18SM	0.47 (0.05, 0.90)	0.030

* Mixed-effect linear regression models were adjusted for age, sex, BMI, and baseline 25(OH)D concentrations. Levels of metabolites were standardized. Serum 25(OH)D concentrations were log-transformed. Abbreviations: 25(OH)D, 25-hydroxyvitamin D_3_; C16Cer, *N*-palmitoyl-sphingosine (d18:1/16:0); C18Cer, *N*-stearoyl-sphingosine (d18:1/18:0); C16dhCer, *N*-palmitoyl-sphinganine (d18:0/16:0); C18dhCer, *N*-stearoyl-sphinganine (d18:0/18:0); S1P, sphingosine 1-phosphate; C16SM, palmitoyl sphingomyelin (d18:1/16:0); C18SM, stearoyl sphingomyelin (d18:1/18:0).

**Table 4:** Adjusted associations of the changes in sphingolipids with changes in BMI and A1c *.

Metabolites	BMI		A1c	
	β	*p*	β	*p*
C16Cer	0.65	0.068	−0.04	0.419
C18Cer	0.97	0.006	0.05	0.302
C16dhCer	0.57	0.076	0.05	0.284
C18dhCer	0.58	0.033	0.11	0.003
Sphingosine	0.01	0.953	0.08	0.037
S1P	−0.04	0.882	0.09	0.020
C16SM	0.31	0.414	−0.02	0.594
C18SM	0.52	0.116	0.03	0.468

* Mixed-effect linear regressions are adjusted for age and sex. Levels of metabolites are standardized. Standardized β coefficients were presented. Abbreviations: BMI, body mass index; C16Cer, *N*-palmitoyl-sphingosine (d18:1/16:0); C18Cer, *N*-stearoyl-sphingosine (d18:1/18:0); C16dhCer, *N*-palmitoyl-sphinganine (d18:0/16:0); C18dhCer, *N*-stearoyl-sphinganine (d18:0/18:0); S1P, sphingosine 1-phosphate; C16SM, palmitoyl sphingomyelin (d18:1/16:0); C18SM, stearoyl sphingomyelin (d18:1/18:0).

## Supplementary Materials

The following are available online at https://www.mdpi.com/2072-6643/12/4/981/s1, Figure S1: Flow diagram of participants, Figure S2: Effect of vitamin D3 supplementation on serum dihydroceramide levels, Figure S3: Effect of vitamin D3 supplementation on serum sphingosine and 1-phosphate derivate levels, Figure S4. Associations between the changes of ceramides and 25(OH)D concentrations, Figure S5. Associations between the changes of dihydroceramide and 25(OH)D concentrations, Figure S6: Associations between the changes of sphingosine, 1-phosphate derivate and 25(OH)D concentrations, Figure S7: Associations between the changes of sphingomyelin and 25(OH)D concentrations, Figure S8: Sphingolipid metabolism, Table S1: Raw changes in 25(OH)D and sphingolipids, Table S2: Adjusted associations between the changes of sphingolipids and 25(OH)D concentrations at baseline *.

## Abbreviations

Cer: ceramide; RCT, randomized clinical trial; C18Cer, *N*-stearoyl-sphingosine (d18:1/18:0); C18SM, stearoyl sphingomyelin (d18:1/18:0); SM, sphingomyelin; BMI, body mass index; 25(OH)D, 25-hydroxyvitamin D3; CV, coefficient of variation; DEQAS, vitamin D external quality assessment scheme; RP, reverse phase; ESI, electrospray ionization; UPLC, ultra-performance liquid chromatography; SD, standard deviation; ANOVA, analysis-of-variance; C16Cer, *N*-palmitoyl-sphingosine (d18:1/16:0); C16dhCer, *N*-palmitoyl-sphinganine (d18:0/16:0); C18dhCer, *N*-stearoyl-sphinganine (d18:0/18:0); S1P, sphingosine 1-phosphate; C16SM, palmitoyl sphingomyelin (d18:1/16:0); dhCer, dihydroceramide; CerS, ceramide synthases; HNC, head and neck cancer; CNS, central nervous system; VDR, vitamin D receptor; Enpp7, Ectonucleotide pyrophosphatase/phosphodiesterase family member 7; SGMS1, Phosphatidylcholine:ceramide cholinephosphotransferase 1; DES2, Sphingolipid delta(4)-desaturase/C4-hydroxylase DES2; ACER1, Alkaline ceramidase 1; LPP1, Lipid phosphate phosphohydrolase 1.
