# Supplementary material for: Vitamin D3 Supplementation Increases Long-Chain Ceramide Levels in Overweight/Obese African Americans: A Post-Hoc Analysis of a Randomized Controlled Trial
Source: Nutrients. 2020 Apr 2;12(4):981. doi: 10.3390/nu12040981 (PMC7230674; doi:10.3390/nu12040981)
Supplement: Supplementary file 1 [file nutrients-12-00981-s001.pdf]

Table S1. Raw changes in 25(OH)D and sphingolipids.

Metabolites		Placebo	600 IU/day	2000 IU/day	4000 IU/day
25(OH)D	Pre	39.7±14.7	35.0±7.7	39.8±10.7	32.9±10.3
	Post	42.0±12.1	56.5±12.2	90.0±32.2	83.2±21.0
	Changes	2.2±13.0	21.5±11.2	50.2±27.1	50.3±19.6
	p-value	0.501	<0.001	<0.001	<0.001
C16Cer	Pre	0.99±0.31	1.11±0.41	1.03±0.26	0.93±0.16
	Post	0.98±0.28	1.10±0.38	1.05±0.24	1.00±0.23
	Changes	-0.01±0.10	-0.00±0.22	0.01±0.19	0.07±0.18
	p-value	0.766	0.965	0.787	0.164
C18Cer	Pre	1.02±0.43	1.17±0.56	1.03±0.44	0.95±0.22
	Post	0.87±0.39	1.19±0.52	1.10±0.50	1.12±0.41
	Changes	-0.15±0.30	0.02±0.28	0.08±0.29	0.17±0.28
	p-value	0.074	0.784	0.307	0.034
C16dhCer	Pre	1.08±0.50	1.13±0.74	1.24±0.65	1.21±0.54
	Post	1.13±0.39	1.16±0.58	1.22±0.56	1.32±0.56
	Changes	0.05±0.40	0.03±0.38	-0.02±0.56	0.11±0.39
	p-value	0.628	0.735	0.875	0.289
C18dhCer	Pre	1.14±0.77	1.12±0.85	1.08±0.97	1.15±1.08
	Post	0.98±0.99	1.00±0.78	1.38±1.52	1.35±1.29
	Changes	-0.16±1.10	-0.13±0.56	0.30±0.73	0.20±0.92
	p-value	0.557	0.404	0.111	0.406
Sphingosine	Pre	1.05±0.68	0.85±0.37	1.07±0.59	0.92±0.39
	Post	0.90±0.41	0.75±0.36	0.94±0.66	0.72±0.32
	Changes	-0.15±0.55	-0.11±0.59	-0.13±0.44	-0.20±0.45
	p-value	0.307	0.498	0.256	0.094
S1P	Pre	1.08±0.28	1.04±0.25	1.09±0.28	1.11±0.30
	Post	1.05±0.31	0.96±0.25	1.21±0.24	0.98±0.26
	Changes	-0.03±0.27	-0.07±0.25	0.03±0.32	-0.13±0.25
	p-value	0.652	0.290	0.683	0.057
C16SM	Pre	0.99±0.19	1.05±0.23	1.03±0.19	0.98±0.10
	Post	1.00±0.18	1.10±0.23	1.09±0.20	1.04±0.16
	Changes	0.01±0.11	0.05±0.11	0.06±0.08	0.07±0.11
	p-value	0.728	0.095	0.010	0.024
C18SM	Pre	1.01±0.24	1.10±0.35	1.05±0.31	0.95±0.15
	Post	0.95±0.28	1.10±0.33	1.14±0.33	1.08±0.22
	Changes	-0.06±0.23	0.01±0.18	0.10±0.19	0.13±0.17
	p-value	0.326	0.891	0.055	0.008

Table S2. Adjusted associations between sphingolipids and 25(OH)D concentrations at baseline***.**

**Metabolites**	**25(OH)D**	
	**β**	**p**
C16Cer	-0.67	0.150
C18Cer	-0.47	0.233
C16dhCer	-0.58	0.230
C18dhCer	-0.24	0.513
Sphingosine	0.18	0.684
S1P	0.41	0.372
C16SM	**-1.29**	**0.004**
C18SM	-0.78	0.061

[*] Linear regression models were adjusted for age, sex, and BMI. Levels of metabolites were standardized. Serum 25(OH)D concentrations were log transformed. Abbreviations: C16Cer, N-palmitoyl-sphingosine (d18:1/16:0); C18Cer, N-stearoyl-sphingosine (d18:1/18:0); C16dhCer, N-palmitoyl-sphinganine (d18:0/16:0); C18dhCer, N-stearoyl-sphinganine (d18:0/18:0); S1P, sphingosine 1-phosphate; C16SM, palmitoyl sphingomyelin (d18:1/16:0); C18SM, stearoyl sphingomyelin (d18:1/18:0).

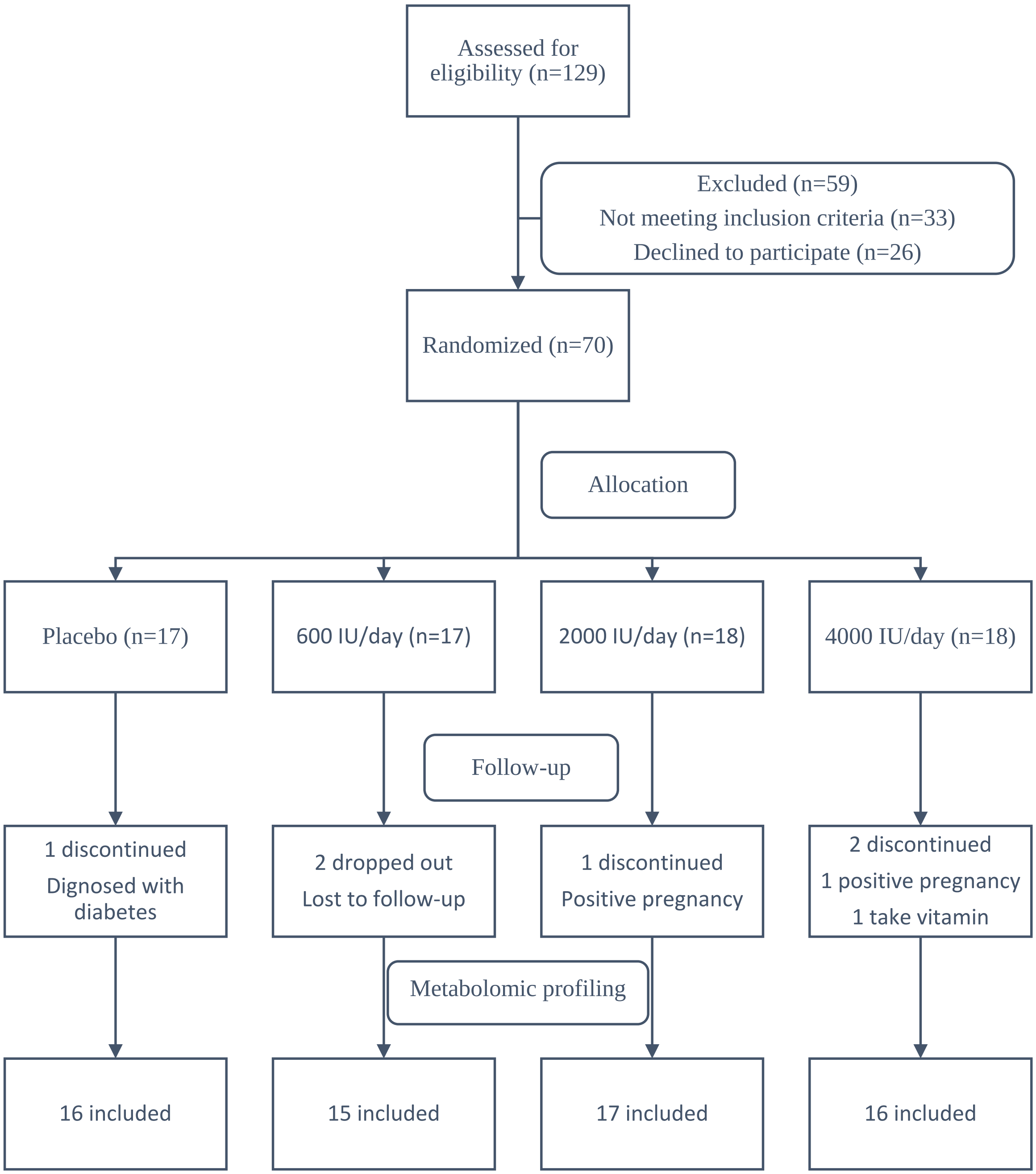

Figure S1. Flow diagram of participants.

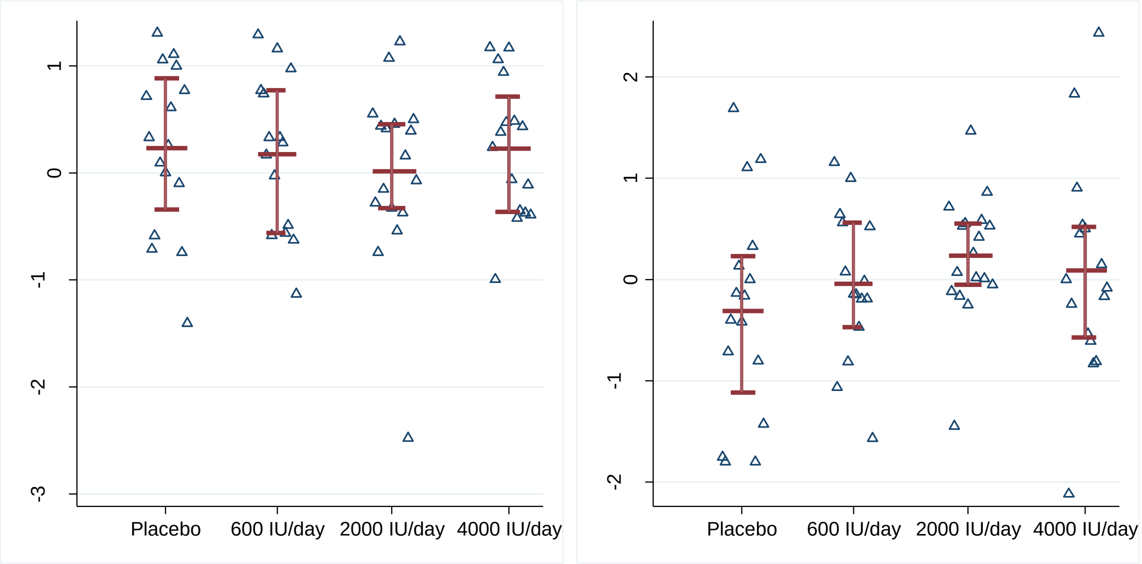

Figure S2. Effect of vitamin D_3 supplementation on serum dihydroceramide levels. Left is C16dhCer, and right is C18dhCer. Y-axis is the change of standardized levels of dihydroceramide (dhCer). Red lines indict 25 percentile, mean and 75 percentile of standardized levels of dhCer in each group. Abbreviations: C16dhCer, N-palmitoyl-sphinganine (d18:0/16:0); C18dhCer, N-stearoyl-sphinganine (d18:0/18:0).

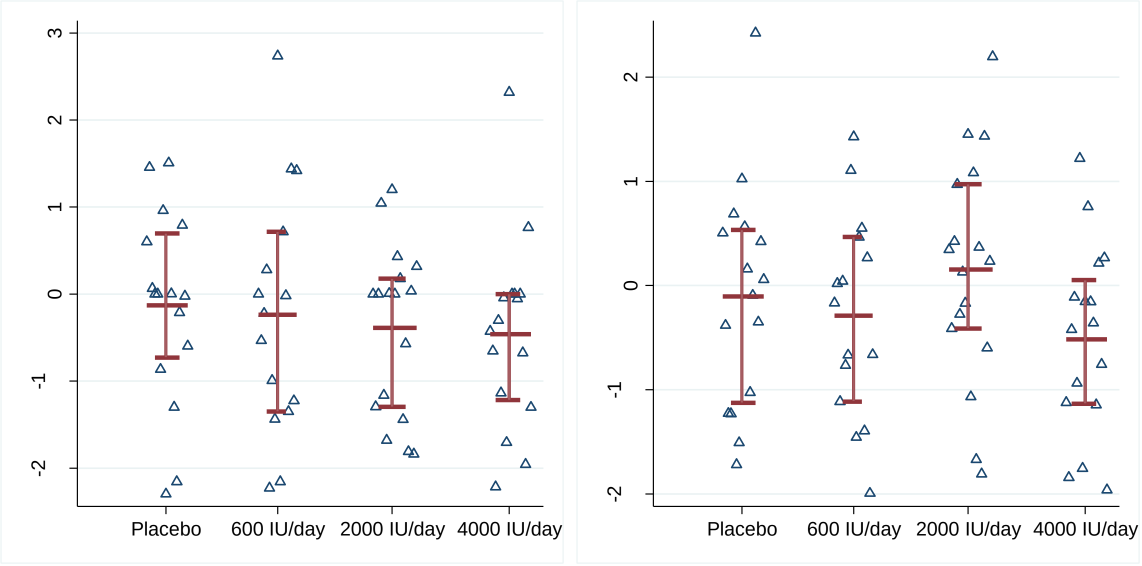

Figure S3. Effect of vitamin D_3 supplementation on serum sphingosine and 1-phosphate derivate levels. Left is sphingosine, and right is S1P. Y-axis is the change of standardized levels of the metabolites. Red lines indicate 25 percentile, mean and 75 percentile of standardized levels of metabolites in each group. Abbreviations: S1P, sphingosine 1-phosphate.

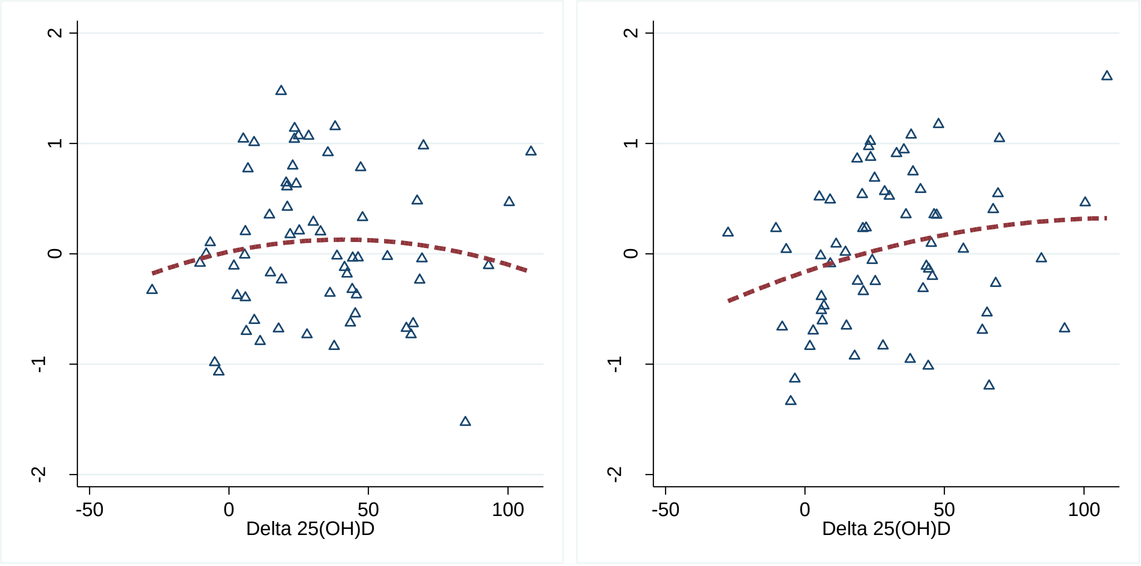

Figure S4. Associations between the changes of ceramides and 25(OH)D concentrations. Left is C16Cer, and right is C18Cer. Y-axis is the change of standardized levels of Cer. Red line is the quadratic prediction. Abbreviations: C16Cer, N-palmitoyl-sphingosine (d18:1/16:0); C18Cer, N-stearoyl-sphingosine (d18:1/18:0).

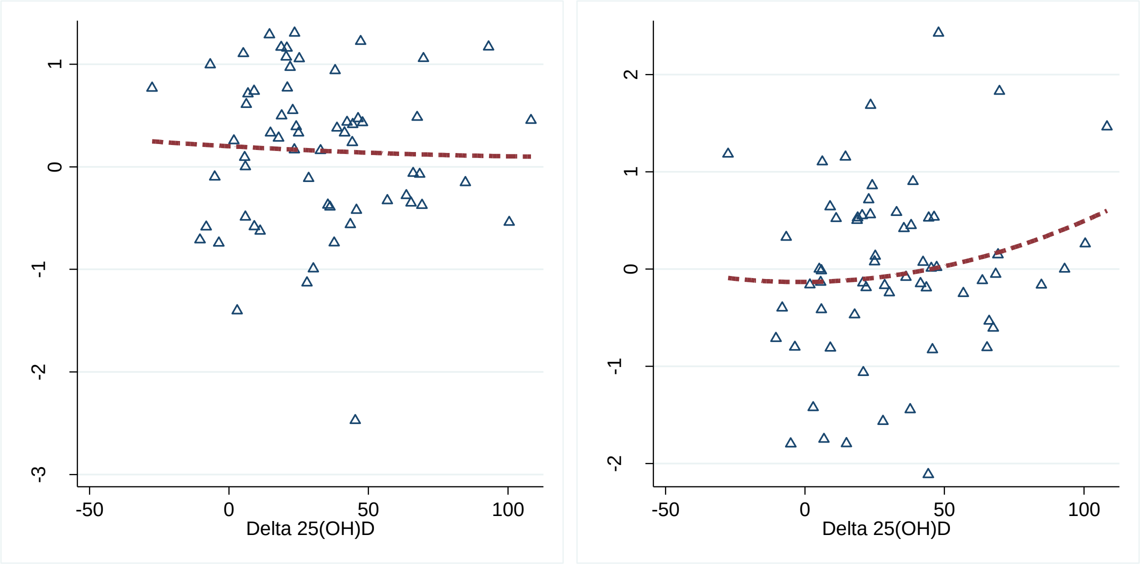

Figure S5. Associations between the changes of dihydroceramide and 25(OH)D concentrations. Left is C16dhCer, and right is C18dhCer. Y-axis is the change of standardized levels of dihydroceramide (dhCer). Red line is the quadratic prediction. Abbreviations: C16dhCer, N-palmitoyl-sphinganine (d18:0/16:0); C18dhCer, N-stearoyl-sphinganine (d18:0/18:0).

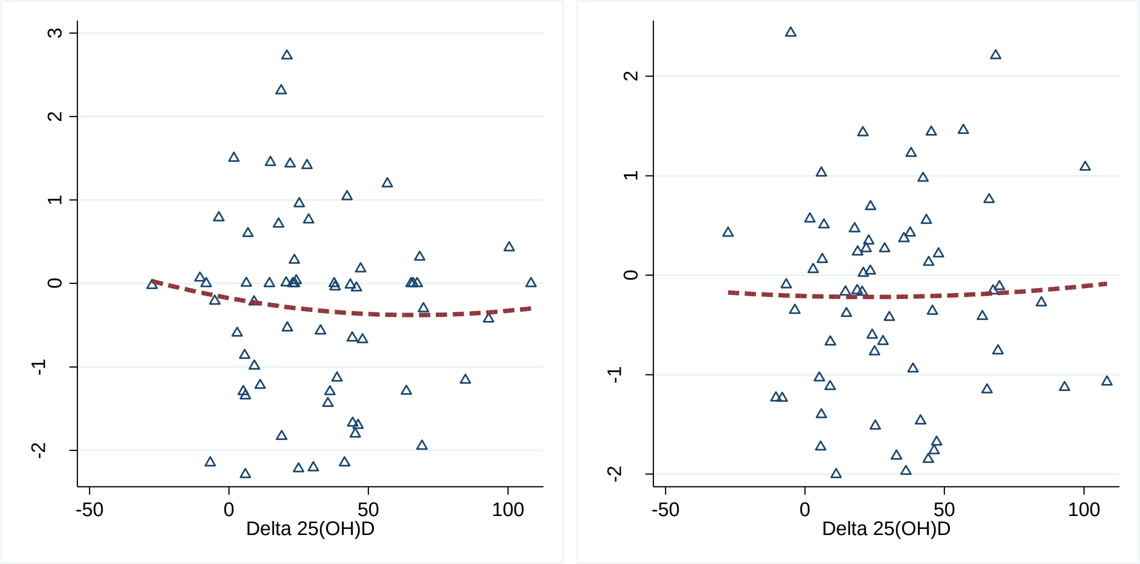

Figure S6. Associations between the changes of sphingosine, 1-phosphate derivate and 25(OH)D concentrations. Left is sphingosine, and right is S1P. Y-axis is the change of standardized levels of Cer. Red line is the quadratic prediction. Abbreviations: S1P, sphingosine 1-phosphate.

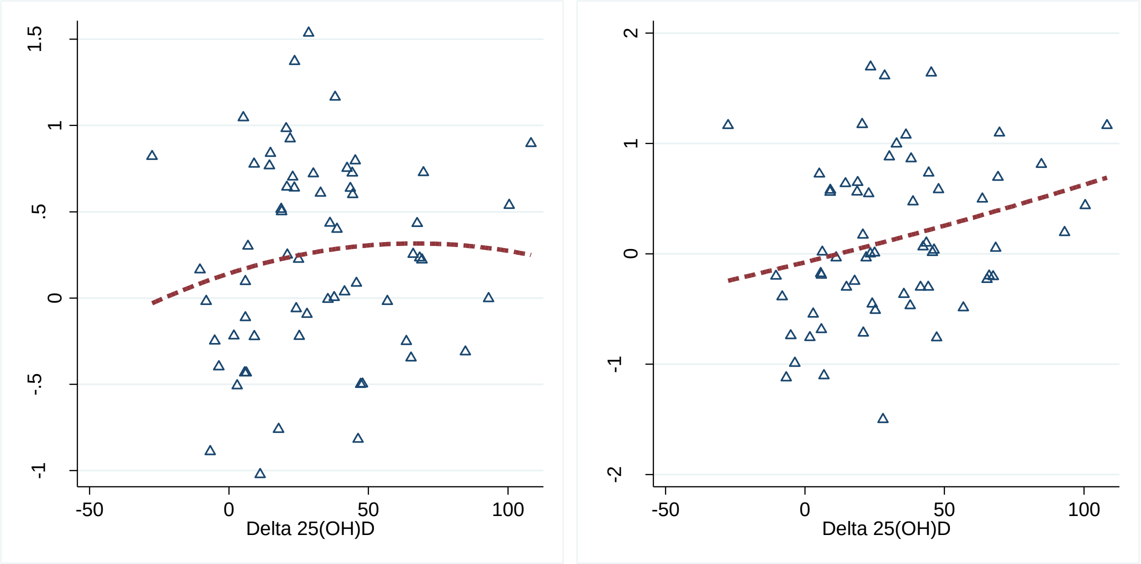

Figure S7. Associations between the changes of sphingomyelin and 25(OH)D concentrations. Left is C16SM, and right is C18SM. Y-axis is the change of standardized levels of SM. Red line is the quadratic prediction. Abbreviations: C16SM, palmitoyl sphingomyelin (d18:1/16:0); C18SM, stearoyl sphingomyelin (d18:1/18:0).

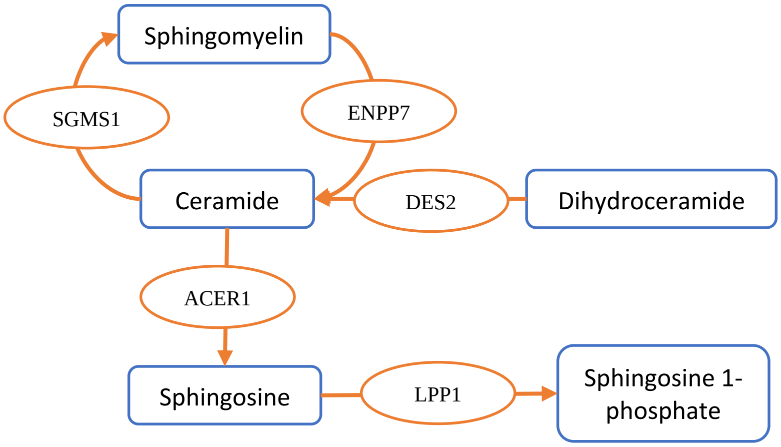

Figure S8. Sphingolipid metabolism. Dihydroceramide (dhCer) is the precursor of Cer, and can be synthesized through N-acylation dihydrosphingosine by one of six ceramide synthases (CerS1-CerS6), each using specific acyl chains. dhCer then dehydrogenated to Cers by dihydroceramide desaturase 2 (Des2). Sphingosine and sphingosine 1-phosphate (S1P) are the degradation products of Cers by alkaline ceramidase 1 (Acer1). Cer and SM are able to transform to each other with the help of ectonucleotide pyrophosphatase/phosphodiesterase family member 7 (Enpp7), and phosphatidylcholine: ceramide cholinephosphotransferase 1 (SGMS1). Abbreviations: Enpp7, Ectonucleotide pyrophosphatase/phosphodiesterase family member 7; SGMS1, Phosphatidylcholine:ceramide cholinephosphotransferase 1; DES2, Sphingolipid delta(4)-desaturase/C4-hydroxylase DES2; ACER1, Alkaline ceramidase 1; LPP1, lipid phosphate phosphohydrolase 1.
